# Comorbidity and survival among women with ovarian cancer: evidence from prospective studies

**DOI:** 10.1038/srep11720

**Published:** 2015-06-29

**Authors:** Yi-Sheng Jiao, Ting-Ting Gong, Yong-Lai Wang, Qi-Jun Wu

**Affiliations:** 1 Department of Obstetrics and Gynecology, Shengjing Hospital of China Medical University, Shenyang, China; 2 Department of Clinical Epidemiology, Shengjing Hospital of China Medical University, Shenyang, China

## Abstract

The relationship between comorbidity and ovarian cancer survival has been controversial so far. Therefore, we conducted a meta-analysis to summarize the existing evidence from prospective studies on this issue. Relevant studies were identified by searching the PubMed, EMBASE, and ISI Web of Science databases through the end of January 2015. Two authors independently performed the eligibility evaluation and data abstraction. Random-effects models were used to estimate summary hazard ratios (HRs) and 95% confidence intervals (CIs) for overall survival. Eight prospective studies involving 12,681 ovarian cancer cases were included in the present study. The summarized HR for presence *versus* absence of comorbidity was 1.20 (95% CI = 1.11–1.30, n = 8), with moderate heterogeneity (*I*^2^ = 31.2%, *P* = 0.179). In addition, the summarized HR for the highest compared with the lowest category of the Charlson’s comorbidity index was 1.68 (95% CI = 1.50–1.87, n = 2), without heterogeneity (*I*^2^ = 0%, *P* = 0.476). Notably, a significant negative impact of comorbidity on ovarian cancer survival was observed in most subgroup analyses stratified by the study characteristics and whether there was adjustment for potential confounders. In conclusion, the findings of this meta-analysis suggest that underlying comorbidity is consistently associated with decreased survival in patients with ovarian cancer. Comorbidity should be taken into account when managing these patients.

Epithelial ovarian cancer (EOC) is the third most common malignancy worldwide among gynecologic cancers, with almost 0.23 million new cases diagnosed in 2012^1^. Since effective screening programs are still lacking, patients with EOC are always diagnosed at advanced stages. Consequently, this cancer caused more than 0.15 million deaths worldwide in 2012^1^. Notably, EOC is the most lethal gynecological malignancy in developed countries^1^, with a 5-year survival rate of 44% based on data from the Surveillance, Epidemiology, and End Results (SEER) program registries^2^. During the past decade, the outcomes of many EOC patients have improved with primary treatments (e.g., surgery and some forms of chemotherapy), but long-term survival of these patients has still not been satisfactory^3^. Given this, there is an urgent need for identifying modifiable prognostic factors that may improve more targeted therapeutic regimens for this disease.

Age, tumor stage and grade, and residual tumor are well-established prognostic factors for the survival of patients with EOC^4^. Meanwhile, previous cohort studies have provided conflicting evidence indicating comorbidity, which is defined as the presence of one or more diseases in addition to the primary disease, as a prognostic factor for the survival of EOC patients^5^. Some studies^6^,^7^,^8^,^9^,^10^ have reported an increased risk of mortality with comorbidity, while others^11^,^12^,^13^ have found no association. Additionally, to our knowledge, the aforementioned studies have not been systematically reviewed. Therefore, to summarize results and clarify the association between comorbidity and the survival of EOC patients, we conducted the present meta-analysis based on published prospective studies.

## Results

### Search results and study characteristics

**Figure 1:**
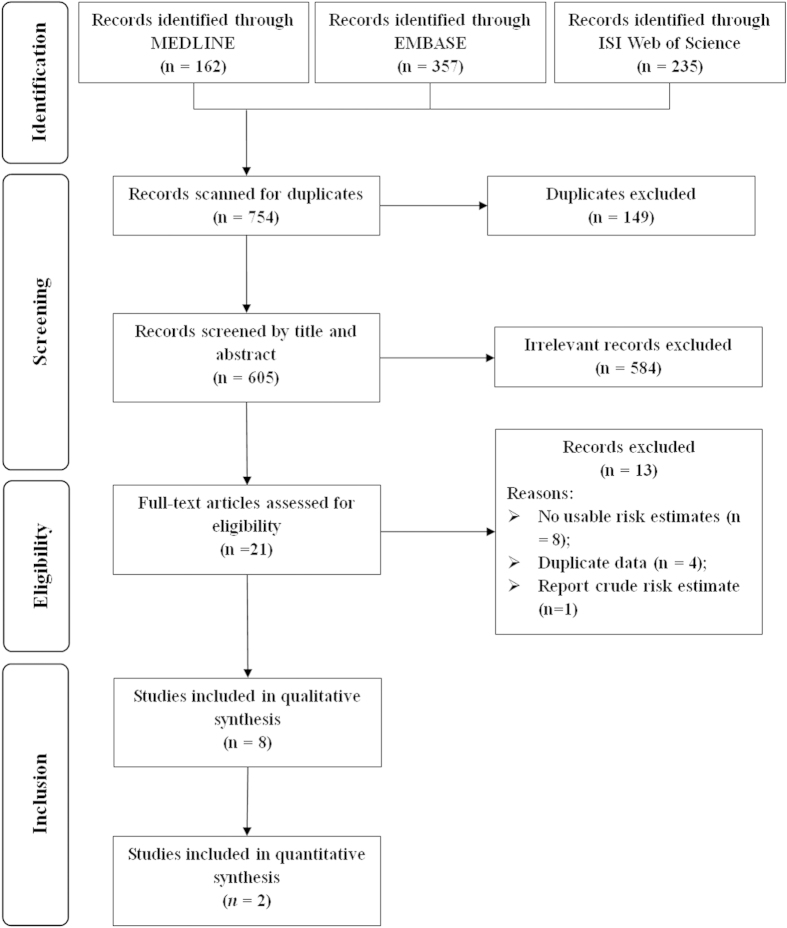
Flow-chart of study selection.

Our systematic literature search in three databases identified 754 articles for eligibility. After initial screening, 149 were excluded as duplicates, 584 by title and abstract scan, and 21 by full-text assessment. Subsequently, thirteen articles were excluded after further evaluation of the full text and for following reasons. Four articles^14^,^15^,^16^,^17^ were replaced by updated or more informative studies, and eight articles^18^,^19^,^20^,^21^,^22^,^23^,^24^,^25^ did not present usable results, while one article^26^ reported the crude risk estimate without adjustment for any potential confounders or established prognostic factors. In the end, a total of 8 articles^6^,^7^,^8^,^9^,^10^,^11^,^12^,^13^ met our inclusion criteria for the final meta-analysis (Fig. 1).

Table 1 provides characteristics of the included studies which were published between 1997 and 2014. Eight studies comprising 12,681 patients with EOC reported an association between comorbidity and overall survival of ovarian cancer. Three studies were carried out in the United States^6^,^10^,^12^, two in Denmark^7^,^8^, and one study each in Australia^13^, Germany^9^, and the Netherlands^11^. Sample size of the included studies ranged from 137 to 5213. All included studies adjusted for age and tumor stage. Half of the included studies adjusted for tumor grade (n = 4) and tumor histology (n = 4). However, fewer included studies adjusted for cancer treatment (n = 2), ascites (n = 2), and residual tumor (n = 2).

### Comorbidity and EOC survival

**Figure 2:**
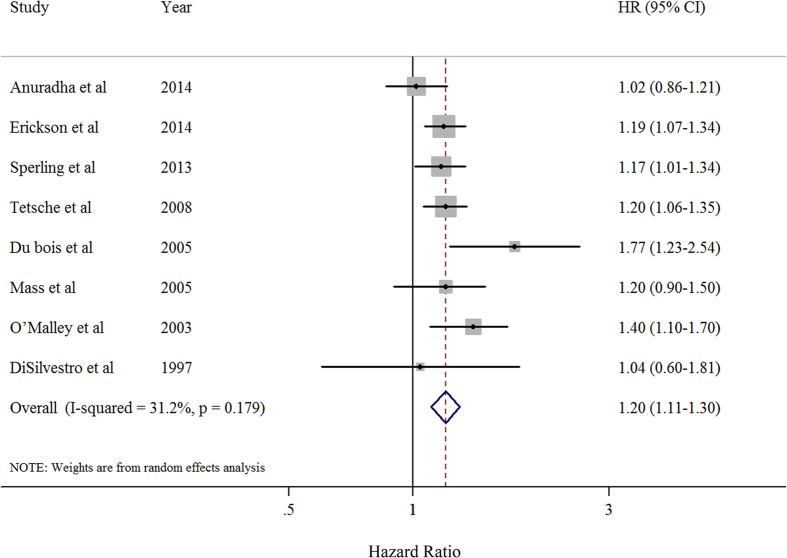
Forest plot (random-effects model) of hazard ratios and 95% confidence intervals of individual studies and pooled estimate for death in patients with ovarian cancer who had comorbidity compared with those without comorbidities. Squares indicate study-specific hazard ratios (size of the square reflects the study-specific statistical weight); horizontal lines indicate 95% CIs; diamond indicates the summary hazard ratio estimate with its 95% CI. HR: hazard ratio.

Figure 2 shows the study-specific and summarized hazard ratios (HRs) and 95% confidence intervals (CIs) of EOC survival for the presence *versus* absence categories of comorbidity. Overall, the summarized analysis showed an HR of 1.20 (95% CI = 1.11–1.30) with moderate heterogeneity (*P* = 0.179, *I*^2^ = 31.2%). There was no evidence of publication bias, both quantitatively (*P* = 0.448 for Egger’s test and *P* = 0.902 for Begg’s test) and qualitatively, on visual inspection of the funnel plot (Supplementary Figure S1).

In addition, two studies were available for the analysis of the highest *versus* the lowest categories of CCI^8^,^13^. The results showed poorer survival among the highest CCI group compared with the lowest CCI women with EOC (HR = 1.68, 95% CI = 1.50–1.87), without heterogeneity (*P* = 0.476, *I*^2^ = 0%).

### Subgroup and sensitivity analysis

To explore the heterogeneity between studies of comorbidity and EOC survival, we performed the stratified and sensitivity analyses. The results of stratified analyses for the association between comorbidity and EOC overall survival are given in Table 2. When the analysis was stratified according to geographic location, the summarized HRs of studies from North America, Europe, and Australia were 1.23 (95% CI = 1.11–1.36), 1.23 (95% CI = 1.10–1.38), and 1.02 (95% CI = 0.86–1.21), respectively. Additionally, similar significant results were observed in the stratified analysis by study period and adjustment for potential confounders (Table 2).

A sensitivity analysis omitting one study at a time and calculating the summarized HRs for the remainder of the studies showed that the 8 study-specific HRs ranged from a low of 1.18 (95% CI = 1.09–1.27; *P* = 0.244; *I*^2^ = 24.3%) after omitting the study by O’Malley *et al.*^10^ to a high of 1.22 (95% CI = 1.14–1.31; *P* = 0.368; *I*^2^ = 7.9%) after omitting the study by Anuradha *et al.*^13^.

## Discussion

To our knowledge, this is first meta-analysis that addressed the role of comorbidity in predicting survival of EOC. The findings of the present study showed that EOC patients with comorbidity had a worse survival than patients without comorbidity. Additionally, survival in patients with EOC may decrease with higher CCI scores. Clinicians are urged to evaluate and reduce any underlying comorbidity carefully in the management of patients with EOC.

It has been proposed that comorbidity in cancer patients may influence the choice of primary treatment (e.g., surgery and chemotherapy) as well as the tumor stage at diagnosis, which in turn affects cancer prognosis and survival^8^,^21^. Compared to EOC patients with comorbidity, patients without comorbidity were more likely to receive standard treatment (primary surgery in combination with chemotherapy)^11^. Additionally, several studies indicated that patients with comorbidity might have lower tolerance to the adjuvant chemotherapy and that there was a possible interaction between the drugs used to treat comorbid diseases and chemotherapy regimens^21^,^27^. Moreover, Tetsche *et al.*^8^ found that the presence of severe comorbidity was associated with an advanced stage of EOC and proposed that patients with comorbidities could experience delay in diagnosis, resulting in a more advanced cancer stage^8^. However, only one study^8^ carried out a stratified analysis according to FIGO stage, and therefore, whether the impact of comorbidity on ovarian cancer survival varies by tumor stage or grade needs further investigation.

This is the first meta-analysis evaluating the association between comorbidity and survival following EOC diagnosis. By combining the evidence from these published prospective studies, we could detect weaker associations than in the individual studies because of the increased statistical power. Additionally, a number of subgroup and sensitivity analyses were carried out to explore the source of heterogeneity. Of note, the results were robust in the aforementioned analyses (Table 2). However, the impact of comorbidity on survival of EOC patients should be quantified by identifying the proportion of these patients in which comorbid disease was the primary cause of death^28^, considering the difficulty in determining causality in this volatile clinical setting, we evaluated the association between comorbidity and EOC survival by comparing survival in patients with and without comorbidity^28^. Thus, several limitations of this study should be acknowledged for the interpretation of our findings.

First, a meta-analysis cannot control for confounders for which there was no adjustment in the primary analysis of individual studies. We excluded the study of Elit *et al.*^26^ because they reported the crude risk estimates without adjustment for any potential confounders. In contrast, all included studies had been adjusted for at least two potential confounders in their primary analyses. Furthermore, we cannot exclude the possibility of unmeasured or residual confounding as a potential explanation for the observed associations. Several studies demonstrated that severe comorbidity was associated with older age, higher tumor stage, performance status, and cancer treatment^7^,^8^. Although we found that the associations were robust in analyses stratified by adjustment for these aforementioned confounding factors (Table 2), less than half the studies adjusted for tumor grade and histology and only two studies adjusted for cancer treatment or residual tumor. Therefore, further studies stratified by established or additional prognostic factors are warranted to better rule out potential effects of residual confounding.

Second, the use of administrative databases, instead of medical records, has been criticized for lacking the accuracy required for research^7^. According to the prevalence of the comorbid illness and characteristic of the administrative databases, studies may translate different comorbidity into CCI. For example, because of the treatment progress of ulcer disease and low prevalence of liver disease in the Danish populations^7^, these two diseases were not registered in their database. On the other hand, CCI has been shown to have a high specificity but relatively low sensitivity^29^. Applying the CCI to administrative databases could introduce misclassifications of comorbidity. Since comorbidity is often underreported and assumed to be homogenous across outcome groups^7^, this misclassification could be non-differential and bias the association between comorbidity and EOC survival toward the null^30^, which implies that our results are likely to be conservative estimates of the true underlying association.

Third, although this meta-analysis summarized the results for the highest *versus* the lowest categories of CCI, the cogency of the evidence is limited by this comparison being available from only two studies. Notably, it would be important to identify the individual comorbid diseases that have the greatest impact on the survival of EOC patients. A recent study using data from the National Cancer Register of Sweden suggested that thromboembolism, hematologic complications and infections have a pronounced effect on survival in women with EOC^19^. Hence, future studies should provide more detail of the association between specific comorbid diseases and survival of EOC, as well as measuring and analyzing the comorbidity as a continuous approach.

Finally, we pre-defined the outcome as overall survival, which was evaluated in the analyses, as opposed to EOC-specific survival. However, EOC patients generally died from their cancer disease, and thus, overall survival was a good surrogate for cancer-specific survival. Besides, although we employed three large databases and checked the references of included studies, a limited number of studies were included in this meta-analysis, and thus, some of the stratified analyses were difficult to conduct, possibly being less reliable, which restricted the interpretation of these findings.

In conclusion, the results from this meta-analysis, based on prospective studies, suggest that comorbidity has a negative impact on EOC survival. The status and severity of comorbid diseases should be taken into consideration by clinicians when making decisions on EOC management. Further studies need to figure out which individual comorbid diseases may have the greatest impact on survival of EOC patients.

## Materials and Methods

### Search strategy

We followed the guidelines developed by the Meta-analysis Of Observational Studies in Epidemiology group (MOOSE) in this meta-analysis^31^. Two independent investigators systematically searched the MEDLINE (PubMed; http://www.ncbi.nlm.nih.gov/pubmed), EMBASE, and ISI Web of Knowledge databases for eligible prospective studies through the end of January, 2015 without limitations. The following terms were used in the search procedure: (comorbidity) and (ovary or ovarian) and (cancer or neoplasm or tumor or carcinoma) and (survival or mortality or prognosis or recurrence). In addition, the reference lists of retrieved articles were carefully hand-searched for additional publications.

### Study selection criteria

Eligibility of studies for inclusion was assessed independently by two investigators. Studies were eligible for inclusion if all the following criteria were fulfilled: (1) the study used a prospective study design; (2) the exposures were defined as comorbidity or Charlson’s comorbidity index (CCI) (Supplementary Table S1); (3) the outcome was defined as overall survival among women with EOC; and (4) there were estimates of the relative risks (RRs) or HRs with 95% Cis, or data was provided for their calculation. If multiple articles were based on the same study population, the most recent report or the report with the most applicable estimates was selected for our analysis. The study reported by Elit *et al.*^26^ was excluded because they provided risk estimates without adjustment for any potential confounders.

### Data extraction

From each study, the following information was extracted in a standardized manner by the two independent investigators: first author’s last name, publication year, geographic location(s) and age of the patients studied, study sample size, study period, whether using CCI to assess the exposure, survival rate, and factors for which adjustment was made in the primary analysis. From each study, we extracted the risk estimates that reflected the greatest degree of control for potential confounders. Differences in data extraction between investigators were uncommon and were resolved by consensus. Similar to our previous study^32^,^33^,^34^,^35^, for studies^8^,^13^ that did not report the results for the presence *versus* absence category of comorbidity, we used the effective-count method proposed by Hamling *et al.*^36^ to recalculate HRs.

### Statistical analysis

In this meta-analysis, we used the random-effects model^37^ to calculate summary HRs and 95% CIs for the presence *versus* absence of comorbidity and the highest *versus* lowest categories of CCI. Statistical heterogeneity between studies was assessed using *I*^2^-statistics^38^. Small study effects, such as publication bias, were evaluated via funnel plots and Egger’s^39^ and Begg’s^40^ tests, where potential small-study bias was considered when P < 0.10. Pre-specified sensitivity analysis was performed by deleting each study in turn to determine the influence of each individual data set on the overall estimate. We also conducted pre-planned stratified analyses according to potentially relevant factors to investigate possible sources of heterogeneity between studies. For all tests, a probability level <0.05 was considered statistically significant. All statistical analyses were conducted by using Stata version 12 software (StataCorp, College Station, TX).

## Additional Information

**How to cite this article**: Jiao, Y.-S. *et al.* Comorbidity and survival among women with ovarian cancer: evidence from prospective studies. *Sci. Rep.* **5**, 11720; doi: 10.1038/srep11720 (2015).

## Supplementary Material

Supplementary Information

## Figures and Tables

**Table 1:** Characteristics of studies of comorbidity and ovarian cancer survival.

First author, (reference), year, country	Age (Year)	No. of Cases	Study period	CCI assessment	Survival rate (%)	Adjusted factors						
						Age	Stage	Grade	Histology	Treatment	Ascites	Residual tumor
Anuradha *et al.*^13^, 2014, Australia	≥18	1192	2005–2012	√	7-year (31%)	√	√	√	√	—	√	—
Erickson *et al.*^6^, 2014, USA	N/A	367	2004–2009	√	N/A	√	√	√	—	—	—	√
Sperling *et al.*^7^, 2013, Denmark	All	3129	2005–2011	√	Overall (46.5%)	√	√	—	√	—	—	√
Tetsche *et al.*^8^, 2008, Denmark	>15	5213	1995–2003	√	Overall (32.4%)	√	√	—	—	—	—	—
Du bois *et al.*^9^, 2005, Germany	All	476	2001–2003	—	Overall (69.1%)	√	√	√	√	—	√	—
Mass *et al.*^11^, 2005, Netherlands	All	1116	1995–2004	√	Overall (42%)	√	√	—	—	√	—	—
O’Malley *et al.*^10^, 2003, USA	All	1051	1994–2001	√	Overall (36.1%)	√	√	√	√	√	—	—
DiSilvestro *et al.*^12^, 1997, USA	All	137	1987–1992	√	4-year (51%)	√	√	—	—	—	—	—

CCI, Charlson’s comorbidity index; N/A, not available.

**Table 2:** Summary risk estimates of the association between comorbidity and ovarian cancer survival.

	No. of studies	Summary HR (95% CIs)	*I*^2^ value (%)	*P*_h_*
Overall	8	1.20 (1.11–1.30)	31.2	0.179
Subgroup analyses				
Geographic location				
North America	3	1.23 (1.11–1.36)	1.9	0.361
Europe	4	1.23 (1.10–1.38)	32.7	0.216
Australia	1	1.02 (0.86–1.21)	N/A	N/A
Study period (years)				
≥7	4	1.19 (1.05–1.34)	43.0	0.154
<7	4	1.23 (1.08–1.39)	37.6	0.186
Adjustment for potential confounders or prognostic factors				
Age at diagnosis				
Yes	8	1.20 (1.11–1.30)	31.2	0.179
No	0	N/A	N/A	N/A
Tumor stage				
Yes	8	1.20 (1.11–1.30)	31.2	0.179
No	0	N/A	N/A	N/A
Tumor grade				
Yes	4	1.26 (1.05–1.50)	69.5	0.020
No	4	1.19 (1.09–1.29)	0	0.961
Tumor histology				
Yes	4	1.25 (1.04–1.51)	69.8	0.019
No	4	1.19 (1.10–1.29)	0	0.969
Cancer treatment				
Yes	2	1.31 (1.12–1.55)	0	0.368
No	6	1.18 (1.08–1.29)	36.7	0.162
Residual tumor				
Yes	2	1.18 (1.08–1.19)	0	0.854
No	6	1.23 (1.08–1.40)	50.4	0.073
Three aforementioned factors				
Yes	6	1.21 (1.09–1.35)	49.7	0.077
No	2	1.19 (1.06–1.34)	0	0.620
Four aforementioned factors				
Yes	5	1.22 (1.08–1.38)	59.7	0.042
No	3	1.19 (1.07–1.33)	0	0.883

CI: confidence interval; HR: hazard ratio; N/A: not available.

^*^P value for heterogeneity within each subgroup.
