# Supplementary material for: Comorbidity and survival among women with ovarian cancer: evidence from prospective studies
Source: Sci Rep. 2015 Jun 29;5:11720. doi: 10.1038/srep11720 (PMC4484350; doi:10.1038/srep11720)
Supplement: Supplementary Information [file srep11720-s1.pdf]

Comorbidity and survival among women with ovarian cancer: evidence from prospective studies

Yi-Sheng Jiao, Ting-Ting Gong, Yong-Lai Wang, Qi-Jun Wu

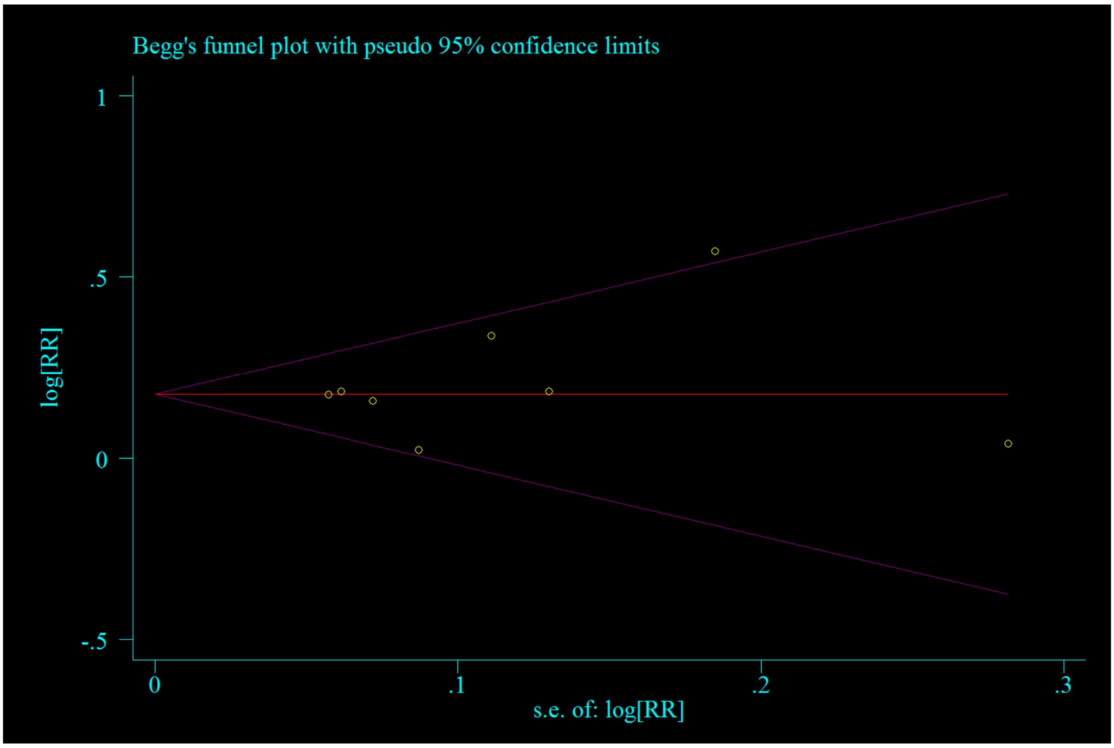

Supplementary Figure S1. Funnel plot corresponding to the random-effects meta-analysis of the relationship between comorbidity and ovarian cancer survival

Supplementary Table S1. Translation of the comorbid conditions into the Charlson Comorbidity Index.

Comorbid conditions	Charlson comorbidity index score
Myocardial infarction	1
Congestive heart failure	1
Peripheral vascular disease	1
Cerebrovascular disease	1
Dementia	1
Chronic pulmonary disease	1
Connective tissue disease	1
Ulcer disease	1
Mild liver disease	1
Diabetes type 1 or type 2	1
Hemiplegia	2
Moderate or severe renal disease	2
Diabetes with end organ damage type 1 or type 2	2
Any tumour (not ovarian cancer)	2
Leukemia	2
Lymphoma	2
Moderate or severe liver disease	3
Metastatic solid tumour	6
AIDS	6
